# Caspase 3 in dying tumor cells mediates post-irradiation angiogenesis

**DOI:** 10.18632/oncotarget.5898

**Published:** 2015-09-29

**Authors:** Xiao Feng, Ling Tian, Zhengxiang Zhang, Yang Yu, Jin Cheng, Yanping Gong, Chuan-Yuan Li, Qian Huang

**Affiliations:** ^1^ The Comprehensive Cancer Center and Shanghai Key Laboratory for Pancreatic Diseases, Shanghai General Hospital, Shanghai Jiao Tong University School of Medicine, Shanghai, China; ^2^ Experimental Research Center, Shanghai General Hospital, Shanghai Jiao Tong University School of Medicine, Shanghai, China; ^3^ The Department of Dermatology, Duke University Medical Center, Durham, NC, USA

**Keywords:** X-irradiation, dying tumor cells, caspase 3, VEGF-A, angiogenesis

## Abstract

Cytotoxic radiotherapy unfavorably induces tumor cells to generate various proangiogenic substances, promoting post-irradiation angiogenesis (PIA), which is one of major causes of radiotherapy failure. Though several studies have reported some mechanisms behind PIA, they have not yet described the beginning proangiogenic motivator buried in the irradiated microenvironment. In this work, we revealed that dying tumor cells induced by irradiation prompted PIA via a caspase 3 dependent mechanism. Proteolytic inactivation of caspase 3 in dying tumor cells by transducing a dominant-negative version weakened proangiogenic effects *in vitro* and *in vivo*. In addition, inhibition of caspase 3 activity suppressed tumor angiogenesis and tumorigenesis in xenograft mouse model. Importantly, we identified vascular endothelial growth factor (VEGF)-A as a downstream proangiogenic factor regulated by caspase 3 possibly through Akt signaling. Collectively, these findings indicated that besides acting as a key executioner in apoptosis, caspase 3 in dying tumor cells may play a central role in driving proangiogenic response after irradiation. Thus, radiotherapy in combination with caspase 3 inhibitors may be a novel promising therapeutic strategy to reduce tumor recurrence due to restrained PIA.

## INTRODUCTION

Radiotherapy is one of the most important strategies for cancer treatment, especially for advanced cancer. Cytotoxic ionizing radiation (IR) induces tumor cell death through various mechanisms [1]. It has already been indicated that besides tumor cell phenotype, endothelial radiosensitivity pivotally modulates tumor response to radiotherapy [2]. Unsurprisingly, IR exerts cytotoxic effects on endothelial cells both *in vitro* [3, 4] and *in vivo* [5, 6]. To some extent, curative effects of radiotherapy are acquired due to vasculature damage and subsequent blockade of blood and nutrient supply. However, contrary to its anti-angiogenic effects, numerous studies have reported that IR would undesirably stimulate tumor cells to up-regulate proangiogenic molecules, such as VEGF [7–10], basic fibroblast growth factor [11], matrix metalloproteinase (MMP)-2 [12, 13], MMP-9 [10, 12], urinary plasminogen activator [10], ephrin-A1 [14], prostaglandin E_2_ [15] and a profile of cytokines [16], which may contribute to tumor radioresistance [7, 8, 14] or tumor repopulation [15, 17].

Additionally, evidence coming from both mouse model [18] and patient specimens [19] has suggested that neovascularization after irradiation mediates tumor recurrence and leads to treatment failure [20]. Therefore, it is of great significance to uncover the mechanisms responsible for PIA. For instance, one group reported that enhanced invasive ability of human microvascular endothelial cells induced by conditioned medium (CM) from irradiated B16 cells was attributable to MMP-2 [13]. Another study discovered that MMP-9 played an important role in PIA, by impelling shedding of Syndecan-1 from cell surface [21]. Furthermore, it was recently reported that depletion of DNA-dependent protein kinase catalytic subunit in glioblastoma cells inhibited IR-induced proangiogenic effects, with decreased secretion of VEGF [22].

Although these studies recognized important mechanisms by which irradiated tumor cells induce and facilitate angiogenesis, they failed to unveil the initial proangiogenic force hidden in the irradiated microenvironment. Since there is a great deal of tumor cell death after radiotherapy, we proposed the hypothesis that irradiation-induced dying tumor cells may serve as a provider, exerting a potent proangiogenic impact on the irradiated milieu. Our data established crucial role of dying tumor cells in promoting PIA. As a deeper step, we unexpectedly found that caspase 3, a well-recognized cysteine protease mediating apoptosis execution, critically modulates proangiogenic effects inflicted by dying tumor cells. We believe that this novel caspase 3-mediated proangiogenic mechanism may provide new therapeutic strategies for cancer treatment or certain irradiation-induced vascular proliferative disorders [23–25].

## RESULTS

### Irradiated HT-29 cells promote human umbilical vein endothelial cell (HUVEC) proliferation and migration *in vitro*

**Figure 1:**
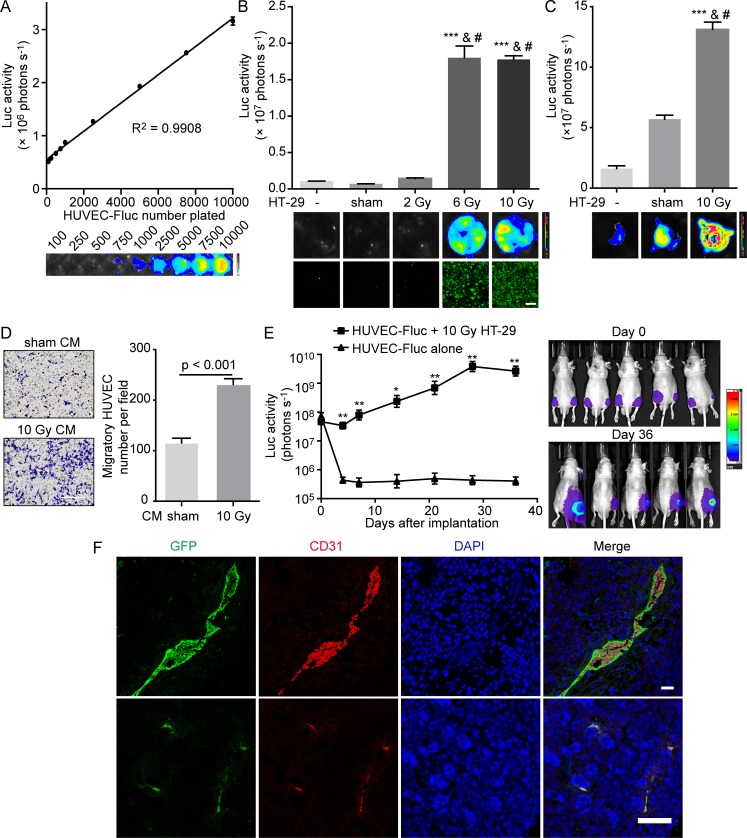
Irradiated HT-29 cells activate HUVECs *in vitro* and support HUVEC survival *in vivo* **A.** Tight correlation (R^2^ = 0.9908) between bioluminescence activity of HUVEC-Fluc and cell number plated. Bottom, representative bioluminescence images showing HUVEC-Fluc seeded at different densities. **B.** Proliferation-stimulating effect of irradiated HT-29 cells on HUVEC-Fluc *in vitro*. Upper panel, proliferation of HUVEC-Fluc cocultured with differentially irradiated HT-29 cells for 14 days was measured by bioluminescence imaging. The differences between higher-dose irradiated groups (6, 10 Gy) and control groups (no feeder, sham irradiation) were highly statistically significant. ***p < 0.001, compared with no feeder. #*p* < 0.001, compared with sham irradiation. *n* = 4. Middle panel, representative bioluminescence images. Lower panel, representative images from confocal microscopy for GFP detection. Scale bar: 250 μm. **C.** Proliferation-stimulating effect of 10 Gy-irradiated HT-29 cells on HUVEC-Fluc seeded in hanging cell culture inserts. Upper panel, proliferation of HUVEC-Fluc in hanging inserts was measured via bioluminescence imaging after a 14-day coculture with different feeders. ***p < 0.001, compared with no feeder. #*p* <0.001, compared with sham-irradiated HT-29 cells. *n* = 3. Lower panel, representative images from bioluminescence imaging. **D.** Left panel, representative images of migratory HUVECs towards different conditioned medium (CM). Scale bar: 250 μm. Right panel, migration of HUVECs to 48-hour CM collected from sham-irradiated and 10 Gy-irradiated HT-29 cells. **E.** Survival-supportive effect of 10 Gy-irradiated HT-29 cells on HUVEC-Fluc *in vivo*. Left panel, quantification of bioluminescence signals. *p < 0.05, **p < 0.01, *n* = 5, co-injection side *versus* HUVEC-Fluc alone. Right panel, representative images of mice undergoing bioluminescence imaging. **F.** Confocal immunofluorescence analysis of tumor sections from co-injection side showing GFP and CD31 coexpression. Scale bar: 25 μm (up); 75 μm (down).

To determine the proangiogenic ability of irradiated tumor cells, we first investigated the influence of irradiated tumor cells on HUVEC proliferation and migration, two initial events required for angiogenesis. To examine whether irradiated tumor cells could stimulate surrounding endothelial cell proliferation, we established the following *in vitro* model. A small number (100-500) of firefly luciferase and green fluorescent protein (GFP) labeled HUVECs, designated as HUVEC-Fluc, were seeded onto a large number (2-2.5 × 10^5^) of HT-29 cells treated with X-irradiation at various doses, described as feeder cells. Proliferation of HUVEC-Fluc was finally measured by bioluminescence imaging after a period of coculture. In addition, to confirm the validity of employing luciferase activity to measure HUVEC-Fluc proliferation, we demonstrated that bioluminescence signals were linearly correlated with HUVEC-Fluc number (Figure 1A). Subsequently, results manifested that HT-29 cells receiving higher-dose irradiation (6 Gray [Gy] and 10 Gy) significantly promoted HUVEC-Fluc proliferation when compared with controls (sham-irradiated feeders and no feeder) (Figure 1B). Notably, the bioluminescence signals of HUVEC-Fluc cocultured with 10 Gy-irradiated HT-29 cells were over 25-fold and 16-fold higher than signals of HUVEC-Fluc cocultured with sham-irradiated HT-29 cells and no feeder, respectively. As a further step, since HUVEC-Fluc were also labeled with GFP in tandem with luciferase, we confirmed the proliferation-stimulating effect of irradiated HT-29 cells on HUVEC-Fluc using confocal microscopy detecting GFP and representative photographs were shown (Figure 1B). Furthermore, our results exhibited that 10 Gy-irradiated HT-29 cells also exerted potent proliferation-stimulating effect on HUVEC-Fluc when HUVEC-Fluc were seeded onto hanging cell culture inserts, hence strongly indicating that soluble transmissible factors secreted from irradiated tumor cells participated in this process (Figure 1C). Apart from great capacity of irradiated tumor cells to promote HUVEC proliferation, we also studied whether irradiated tumor cells could enhance HUVEC migration. CM collected from HT-29 cells exposed to 10 Gy irradiation displayed highly stronger property to promote HUVEC migration, compared with CM from sham-irradiated HT-29 (Figure 1D). Thereby, these results indicate that irradiated tumor cells *in vitro* potently stimulate HUVEC proliferation and migration, in which soluble factors released from irradiated tumor cells may be involved.

### Irradiated tumor cells support HUVEC survival *in vivo*

Continuing our study, we then investigated whether irradiated tumor cells could establish a proangiogenic microenvironment *in vivo*. We injected a small number (1 × 10^5^) of HUVEC-Fluc mixed with a large number (1 × 10^6^) 10 Gy-irradiated HT-29 cells subcutaneously into right hind legs of nude mice. An equal number of HUVEC-Fluc were injected subcutaneously into left hind legs of nude mice as control. Subsequently, we monitored the survival or growth of HUVEC-Fluc *in vivo* noninvasively via bioluminescence imaging. Results showed that bioluminescence signals of left hind legs of nude mice decreased significantly 4 days after injection, indicating that HUVEC-Fluc alone failed to survive *in vivo* (Figure 1E). However, bioluminescence signals of co-injection side were maintained and gradually increased after injection despite a slight decline on day 4, suggesting that irradiated HT-29 cells engendered a proangiogenic microenvironment in which HUVEC-Fluc survived (Figure 1E). Moreover, in order to confirm that HUVEC-Fluc did survive *in vivo*, immunofluorescence staining for GFP and CD31 was carried out to analyze tumor sections from co-injection side. As shown in Figure 1F, not only did GFP and CD31 co-expression exist, but also HUVEC-Fluc formed vessel-like structure, indicating that HUVEC-Fluc participated in vascularization of tumors.

### X-irradiation induces caspase 3 activation in dying tumor cells

**Figure 2:**
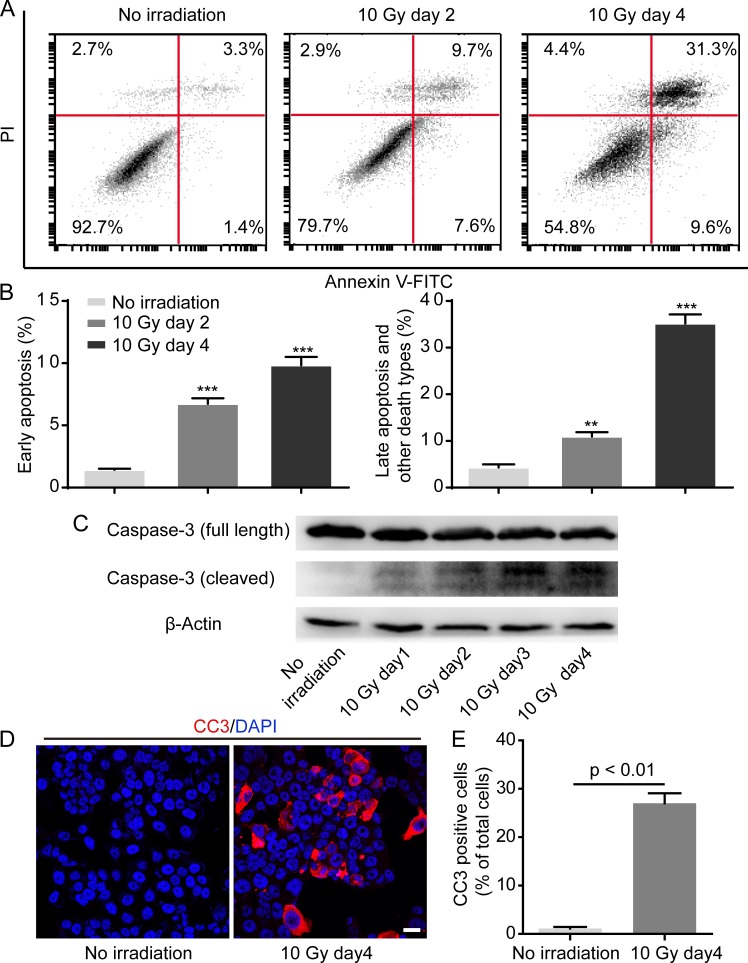
Irradiation induces caspase 3 activation in dying HT-29 cell **A.** Representative flow cytometry plots showing HT-29 cell death in response to 10 Gy irradiation. **B.** Quantification of percentage of early apoptosis and late apoptosis and other death types. **p < 0.01; ***p < 0.001. 10 Gy day 2 or 10 Gy day 4 *versus* no irradiation, *n* = 3. **C.** Western blot analysis showing caspase 3 activation in HT-29 cells in response to 10 Gy irradiation. **D.** Confocal immunofluorescence analysis confirming caspase 3 activation after 10 Gy irradiation. Scale bar: 25 μm. **E.** Quantification analysis depicting that CC3 positive cell percentage was prominently higher on day 4 after 10 Gy irradiation than no irradiation.

It has been generally accepted that ionizing irradiation can bring about tumor cell death [1]. To verify whether HT-29 cell death occurs in reply to X-irradiation, we utilized flow cytometry analysis by means of FITC Annexin V and propidium iodide (PI) double staining. Results showed that FITC Annexin V positive and PI negative (generally considered as early apoptosis) percentage and FITC Annexin V and PI both positive (end stage apoptosis or other death types) percentage of HT-29 cells treated with 10 Gy irradiation significantly increased in a time-dependent manner (Figure 2A, 2B). Remarkably, the percentages of early apoptosis and late apoptosis or other death types were respectively over 6-fold and 8-fold higher on day 4 after 10 Gy irradiation than non-irradiated control (Figure 2B). Because caspase 3 plays a pivotal role in apoptosis execution phase, we next investigated whether caspase 3 was activated in HT-29 cells upon exposure to X-irradiation. Western blot analysis displayed that cleaved caspase 3 (CC3) expression gradually elevated in HT-29 cells after 10 Gy irradiation, indicating that 10 Gy irradiation did lead to caspase 3 activation (Figure 2C). In addition, we also reinforced CC3 expression in HT-29 cells treated with 10 Gy irradiation by means of confocal immunofluorescence analysis (Figure 2D). Prominently, the percentage of CC3 positive cells 4 days after 10 Gy irradiation was over 24-fold in comparison with non-irradiated control (Figure 2E). Overall, these data supported that 10 Gy irradiation did contribute to caspase 3 activation in dying HT-29 cells.

### Proteolytic inhibition of caspase 3 in dying tumor cells mitigates their proangiogenic effects *in vitro* and *in vivo*

**Figure 3:**
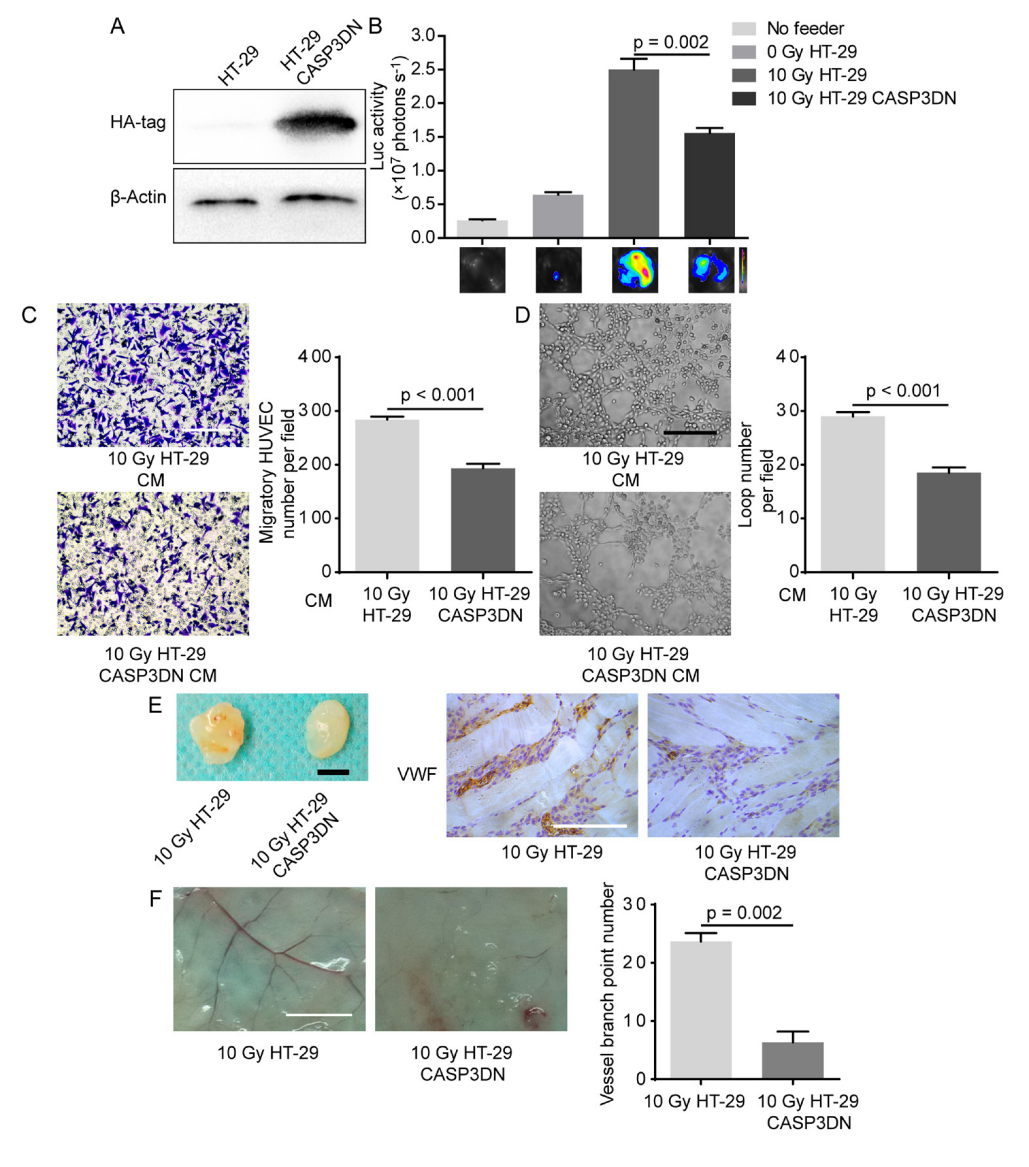
Caspase 3 in dying tumor cell mediates post-irradiation angiogenesis *in vitro* and *in vivo* **A.** Dominant-negative caspase 3 expression was confirmed by Western blot analysis (HA-tag was fused with dominant-negative caspase 3 in tandem). **B.** Proliferation-stimulating effect of dying HT-29 CASP3DN cells was highly inferior to that of dying parental HT-29 cells. **C.** Left panel, representative images of HUVEC migration to different indicated CM. Scale bar: 250 μm. Right panel, quantification of HUVEC migration to indicated CM. **D.** Left panel, representative images of tube formation of HUVECs in different indicated CM. Scale bar: 250 μm. Right panel, loop number quantification of tube formation assay performed in different CM. **E.** Matrigel mixed with 10 Gy-irradiated HT-29 cells or HT-29 CASP3DN cells was subcutaneously injected into flank of nude mice for 8 days. Left panel, representative images of plugs mixed with indicated cells. Scale bar: 5 mm. Right panel, immunohistochemical analysis of plug sections for VWF staining. Scale bar: 125 μm. **F.** Left panel, representative images of skin vasculature adjacent to indicated plugs. Scale bar: 5 mm. Right panel, quantification of dermal blood vessel adjoining indicated plugs.

What is the molecular mechanism underlying proangiogenic role of dying tumor cells? Our previous studies found that caspase 3 in dying tumor cells induced by cytotoxic treatments (radiotherapy or chemotherapy) is responsible for tumor cell repopulation [15, 26, 27], so we wondered whether or not caspase 3 in dying tumor cells also played a driving role in PIA. To examine our hypothesis, we first inhibited proteolytic activity of caspase 3 by transducing a dominant-negative version of caspase 3 (C163A) [28, 29], designated as CASP3DN. Western blot analysis confirmed the expression of CASP3DN in HT-29 cells by detecting the expression of hemagglutinin (HA)-tag (Figure 3A), whose gene had been tandem inserted into the lentiviral vector. Importantly, taking advantage of the formerly-established *in vitro* coculture model, we observed that 10 Gy-irradiated HT-29 CASP3DN cells showed significantly diminished proliferation-promoting effect on HUVEC-Fluc when compared with equally treated HT-29 cells (Figure 3B). Subsequently, we examined the migratory capacity of HUVECs toward CM from irradiated parental HT-29 cells and HT-29 CASP3DN cells. We observed that migration of HUVECs toward CM collected from 10 Gy-irradiated HT-29 CASP3DN cells was significantly inhibited, compared with CM from 10 Gy-irradiated HT-29 cells (Figure 3C). Next, to test the ability of different CM to induce HUVECs to form capillary-like structures, we performed tube formation assay. As shown in Figure 3D, tube formation capacity of HUVECs was greatly weakened when mixed with CM collected from 10 Gy-irradiated HT-29 CASP3DN cells. Taken together, these findings suggested that caspase 3 in dying tumor cells performs a supportive role in promoting surrounding angiogenic processes, including endothelial cell proliferation, migration and tube formation.

Having shown that caspase 3 in dying tumor cells mediates proangiogenic effects *in vitro*, we subsequently explored whether caspase 3 in dying tumor cells mediates proangiogenic response *in vivo* by performing matrigel plug assay. We observed that blood vessel formation in matrigel mixed with 10 Gy-irradiated HT-29 CASP3DN cells was significantly reduced, compared with blood vessel formation in matrigel mixed with equally treated HT-29 cells (Figure 3E). Further immunohistochemical staining of matrigel plug sections for von Willebrand factor (VWF) confirmed our macroscopic observation (Figure 3E). Additionally, our results showed that skin vascularization adjacent to plugs containing 10 Gy-irradiated HT-29 CASP3DN cells was remarkably less than control (Figure 3F). Hence, evidence *in vivo* also indicated that caspase 3 in dying tumor cells plays a supportive role in surrounding angiogenesis.

### Proteolytic inactivity of caspase 3 suppresses tumor angiogenesis and tumorigenesis

**Figure 4:**
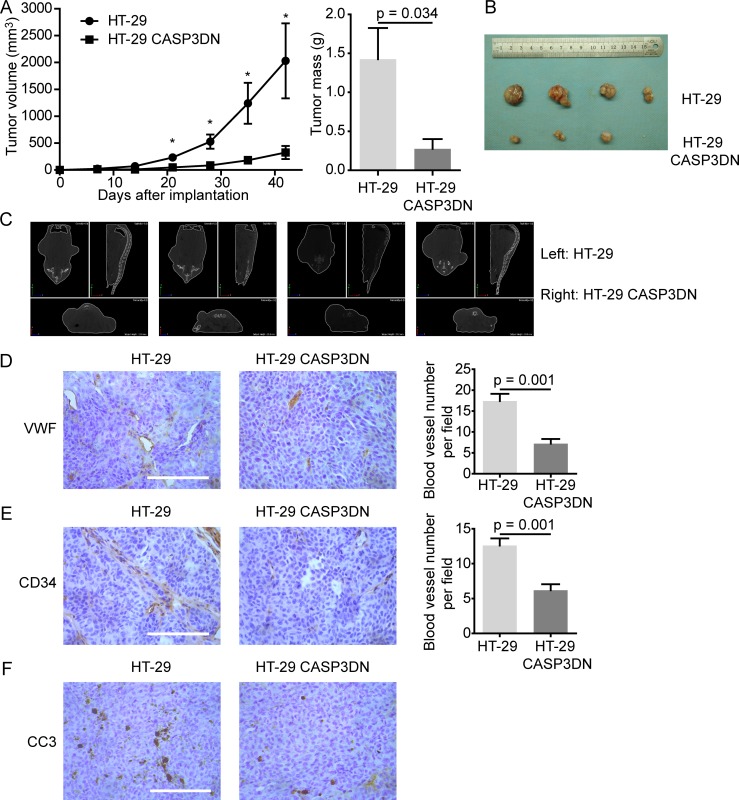
Proteolytic inactivity of caspase 3 restrains tumor angiogenesis and tumorigenesis Equal numbers of HT-29 cells and HT-29 CASP3DN cells were subcutaneously injected into either flank (left: HT-29 cells; right: HT-29 CASP3DN cells) of nude mice. **A.** Tumor growth (volume and mass) was inhibited in HT-29 CASP3DN cells, compared with parental cells. *p < 0.05, *n* = 4. **B.** Photographs showing tumors. **C.** Micro-CT analysis of tumor-bearing mice before sacrificing them. **D. E.** Left panel, immunohistochemical staining of tumor sections for endothelial markers, VWF and CD34. Right panel, quantification of blood vessel number in indicated tumor sections. Scale bar: 125 μm. **F.** Immunohistochemical staining of tumor sections for CC3. Scale bar: 125 μm.

To further illustrate that caspase 3 in tumor cells mediates angiogenesis, we carried out tumor xenograft assay in nude mice using parental HT-29 cells and HT-29 CASP3DN cells in the absence of irradiation. Considering that tumor cell death is common to occur during tumorigenesis [30], we supposed that those naturally dying tumor cells could also promote tumor angiogenesis despite the absence of irradiation. According to our assumption, subcutaneously implanted HT-29 CASP3DN cells, due to dysfunction of caspase 3, would elicit less angiogenesis and therefore diminish tumorigenicity, as compared to parental HT-29 cells. Conforming to our speculation, results demonstrated that HT-29 CASP3DN cells exhibited significantly weakened tumor forming ability both in tumor volume and tumor mass (Figure 4A, 4B). Moreover, results from micro-computed tomography (micro-CT) validated our observations (Figure 4C). What is more important, immunohistochemical analysis of xenograft tumor sections for endothelial markers, including VWF and CD34, revealed that tumor angiogenesis was less in HT-29 CASP3DN cell xenograft than in parental HT-29 cell xenograft (Figure 4D, 4E). Next, consistent with our hypothesis, immunohistochemical staining for CC3 revealed that the CC3 expression was higher in parental HT-29 cell xenograft than in HT-29 CASP3DN cell xenograft (Figure 4F). Thereby, these data suggested that caspase 3 deactivation may curb tumor angiogenesis and therefore inhibit tumor growth.

### VEGF-A induction and secretion in response to X-irradiation is inhibited with caspase 3 inhibition

**Figure 5:**
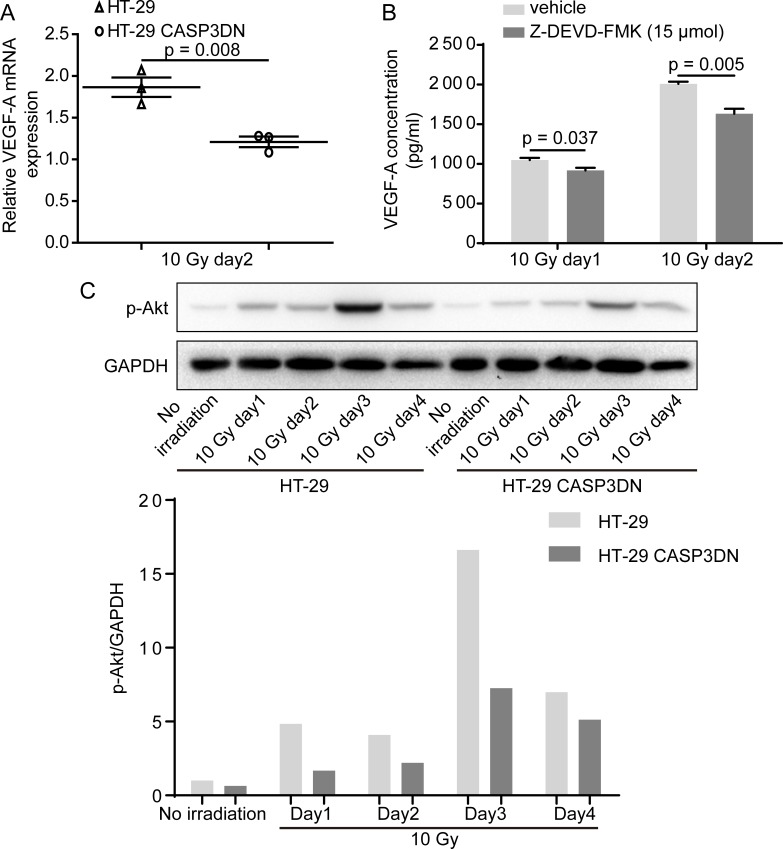
VEGF-A induction and secretion in response to irradiation is inhibited when caspase 3 is proteolytically deactivated **A.** VEGF-A mRNA levels of HT-29 and HT-29 CASP3DN cells 2 days after 10 Gy irradiation were examined by quantitative real-time PCR. **B.** ELISA showing that VEGF-A concentration in supernatant collected from 10 Gy-irradiated HT-29 cells was reduced with administration of a specific caspase 3 inhibitor, Z-DEVD-FMK, at 15 μmol. **C.** Western blot analysis indicating that up-regulation of p-Akt induced by 10 Gy irradiation was repressed in HT-29 CASP3DN cells. Bottom, bar chart displaying relative p-Akt expression normalized to GAPDH.

Our aforementioned results indicated that soluble substances mediate proangiogenic response caused by dying cells, so what are the downstream factors linking caspase 3 with its proangiogenic role? Previous studies have suggested that inhibition of caspase 3 activity suppresses release of VEGF [31, 32], so we wondered whether the impaired proangiogenic effects of irradiated HT-29 CASP3DN cells were attributable to reduction of VEGF. To test our hypothesis, we firstly performed PCR assay to measure the relative expression of VEGF-A. Our results showed that the relative expression of VEGF-A mRNA was lower in HT-29 CASP3DN cells than in HT-29 cells on day 2 since 10 Gy irradiation (Figure 5A). In addition to mRNA level, using enzyme-linked immunosorbent assay (ELISA) we also detected the VEGF-A concentration of cell culture supernatant at protein level. ELISA results showed that VEGF-A concentration of supernatant from 10 Gy-irradiated HT-29 cells with administration of Z-DEVD-FMK, a caspase 3 inhibitor, was significantly lower than that from 10 Gy-irradiated HT-29 cells treated with vehicle control (Figure 5B). Subsequently, another question struck us that how caspase 3, a protease mediating apoptosis, could regulate expression and secretion of VEGF-A. It has been generally considered that Akt signaling has a pivotal role in regulating VEGF expression under multifarious circumstances [33]. To determine whether Akt activation mediates VEGF-A expression regulation by caspase 3, we performed Western blot analysis to detect phosphorylated-Akt (p-Akt) expression in parental HT-29 cells and HT-29 CASP3DN cells in response to irradiation. Intriguingly, we observed that p-Akt induction in response to 10 Gy irradiation was highly restricted in HT-29 CASP3DN cells, in comparison with parental HT-29 cells (Figure 5C).

### VEGF-A blockade represses proangiogenic effects of dying tumor cells

**Figure 6:**
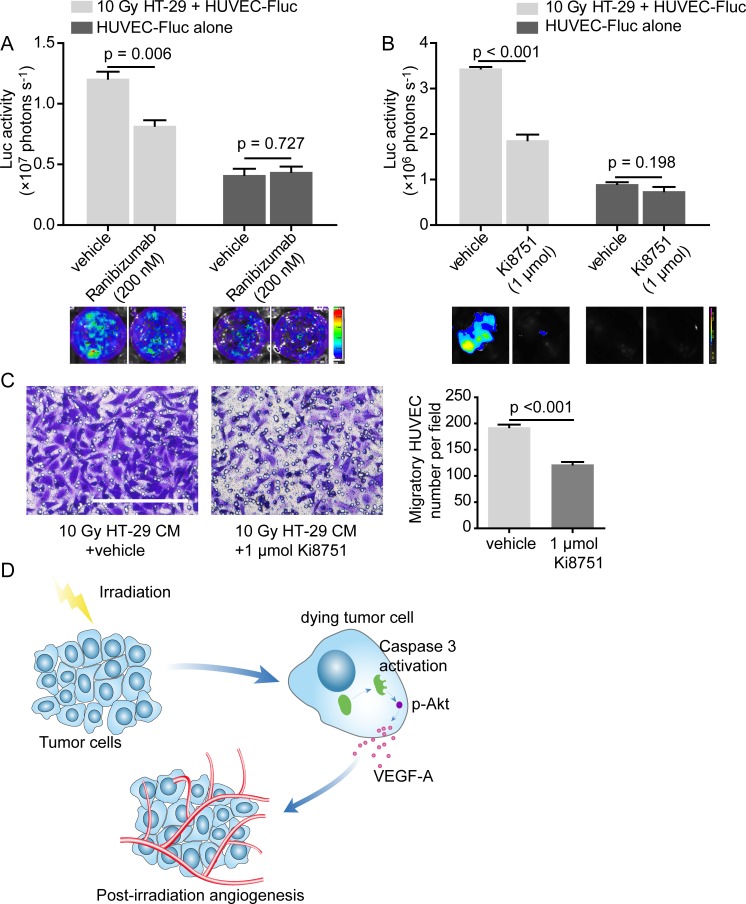
VEGF-A blockade weakens proangiogenic effects of dying HT-29 cells **A.** Ranibizumab (200 nM), a neutralizing antibody against VEGF-A, diminished the proliferation-stimulating effect of dying HT-29 cells on HUVEC-Fluc. **B.** Ki8751 (1 μmol), a VEGFR2 inhibitor, extenuated the proliferation-stimulating effect of dying HT-29 cells on HUVEC-Fluc. **C.** Left panel, migration of HUVEC towards CM from irradiated HT-29 cells was impaired with administration of Ki8751 (1 μmol). Scale bar: 250 μm. Right panel, quantification of the effect of Ki8751 on HUVEC migration to CM from 10 Gy-irradiated HT-29 cells. **D.** Schematic overview summarizing the mechanism of cell death-mediated post-irradiation angiogenesis.

As a further step, in order to substantiate that VEGF-A mediates proangiogenic response induced by dying tumor cells, we investigated whether proangiogenic response attenuated when VEGF-A was blocked. Ranibizumab, a neutralizing antibody against VEGF-A, remarkably damaged the proliferation-stimulating effect of dying HT-29 cells on HUVEC-Fluc, while the concentration of Ranibizumab used did not interfere in HUVEC-Fluc proliferation alone (Figure 6A). Apart from utilizing a neutralizing antibody against VEGF-A, we also pharmacologically tested whether VEGF-A mediated proliferation-promoting effect of dying tumor cells on HUVEC-Fluc. Results showed that Ki8751, a VEGF receptor 2 (VEGFR2) inhibitor, noticeably inhibited proliferation-stimulating effect of dying HT-29 cells and the concentration of Ki8751 did not affect HUVEC-Fluc proliferation alone (Figure 6B). Additionally, Ki8751 of 1 μmol also impaired the migratory ability of HUVEC toward CM collected from 10 Gy-irradiated HT-29 cells (Figure 6C). Accordingly, these findings suggested that VEGF-A is a possible downstream molecule of caspase 3, mediating the proangiogenic effects of dying tumor cells via interacting with VEGFR2.

In summary, based on these results, we finally would like to propose a novel caspase 3 mediated proangiogenic mechanism accounting for PIA, of which a schematic representation was shown (Figure 6D).

## DISCUSSION

During cytotoxic treatments like radiotherapy, tumor cell death occurs. Our findings surprisingly found that dying tumor cells promote PIA through a caspase 3 dependent mechanism. Induction of tumor cell apoptosis has been a vital strategy in cancer therapy, whereas our results counterintuitively suggest that apoptotic tumor cells enhance surrounding angiogenesis, possibly mediating radioresistance or tumor relapse due to the fact that tumor growth is tightly reliable on angiogenesis. In this work, we demonstrated that X-irradiation-induced dying tumor cells markedly enhanced surrounding endothelial cell proliferation and migration and bolstered endothelial cell survival *in vivo*. What is important, our results both *in vitro* and *in vivo* unexpectedly illustrated that caspaes 3, a key executioner of apoptosis, in dying tumor cells mediated their proangiogenic effects. Furthermore, caspase 3 inactivity significantly suppressed tumor angiogenesis in xenograft model and tumor growth. In the end, our results unexpectedly revealed that VEGF-A induction and secretion in response to irradiation was inhibited when caspase 3 was deactivated and VEGF-A blockade attenuated proangiogenic effects of dying tumor cells.

Similar to our findings, one study, though not from cancer research area, has also linked caspase-3 with angiogenesis. The study reported that the proangiogenic effects of lipid-injured hepatocytes are dependent on caspase 3 activity, since inhibition of caspase 3 activity reduces release of proangiogenic microparticles from injured hepatocytes [31]. In addition, though it seems to be paradoxical to link caspase 3, an execution molecule of cell apoptosis, with proangiogenic response, there have been accumulating studies clarifying diverse growth-promoting effects of caspase 3, such as fibrosis [34], osteoclastogenesis [32], tissue regeneration [35], oncogenic transformation [36] and tumor repopulation [15]. Consequently, we would like to propose a bold hypothesis that this novel caspase 3-regulating angiogenesis pathway may be a conservative mechanism involving in various pathophysiological processes like wound healing, fracture healing, tissue regeneration, and tumor repopulation.

In our study, we showed that caspase 3 inactivity extenuated irradiation-induced VEGF-A upregulation. Consistent with our results, another study demonstrated that pharmacologically suppressing caspase 3 activity inhibits fatigue-induced VEGF upreguation in osteocytes. Regarding the potential mechanism accounting for VEGF-A regulation by caspase 3, we demonstrated that Akt activation induced by irradiation was mitigated when caspase 3 was deactivated. Another possible mechanism explaining how caspase 3 modulates VEGF expression may involve protein kinase Cδ (PKCδ). Numerous studies have indicated that caspase 3-dependent PKCδ activation mediates apoptosis under various circumstances [37–41]. Moreover, PKCδ has been reported to tightly regulate VEGF expression [42]. Therefore, it would be worthwhile to determine in future studies whether caspase 3-dependent PKCδ activation mediates proangiogenic effects of dying tumor cells, by regulating VEGF expression.

This study implicates that combination of radiotherapy and caspase 3 inhibitors may act as a more effective strategy for cancer treatment, through mitigating PIA, which is required for tumor regrowth. Interestingly, our previous study has already illustrated caspase 3 inhibition suppresses tumor repopulation during radiotherapy. It is highly possible that alternative mechanism interpreting that caspase 3 inhibition restricts tumor repopulation is due to quelled angiogenesis after irradiation. Indeed, there has been evidence showing that combination of radiotherapy and M867, a selective inhibitor of caspase 3, effectively extends tumor growth delay in mouse xenograft model, with great reduction of vascular density [43].

Furthermore, the VEGF-mediated post-irradiation proangiogenic mechanism revealed in this paper supported the previously proposed notion of therapy-induced tumor progression via VEGF [44]. Indeed, it has been suggested that Avastin (bevacizumab) potentiates chemotherapy by hampering VEGF-mediated reactive resistance to therapy, rather than directly inhibiting tumor angiogenesis [45]. Also, there is evidence suggesting that radiotherapy combined with anti-angiogenic agents (for example, bevacizumab and VEGFR inhibitors) would be a promising therapeutic modality [46, 47]. Considering that p-Akt may mediate irradiation-induced VEGF upregulation, it is conceivable that rapamycin, blocking Akt/mTOR pathway, combined with radiotherapy may serve as a novel treatment strategy for cancer through better targeting tumor vasculature [48, 49].

We therefore hope that this novel caspase 3-mediated proangiogenic pathway could provide more efficacious and multifold therapeutic targets for cancer radiotherapy or even other irradiation-induced vascular proliferative diseases.

## MATERIALS AND METHODS

### Cell culture and cell irradiation

HUVECs were obtained from American Type Culture Collection and cultured in RPMI-1640 (Thermo Fisher Scientific, MA, USA) supplemented with 10% fetal bovine serum (FBS) (Gibco, life technologies, Auckland, NZ). Human colorectal cancer cell line HT-29 was available from the Chinese Academy of Science (Shanghai, China) and grown in Dulbecco's Modified Eagle's Medium (DMEM) (Thermo Fisher Scientific) supplemented with 10% FBS. X-ray irradiation of cells was performed with an Oncor linear accelerator (Siemens, Amberg, Germany), of which the dose rate is about 3.6 Gy/min.

### Gene transduction

To transduce exogenous genes into target cells, we used the pLEX lentiviral vector system obtained commercially from Open Biosystem (Huntsville, AL, USA). The firefly luciferase (Fluc) and green fluorescent protein (GFP) fusion gene [15] was kindly provided by Prof. Chuan-Yuan Li and delivered into HUVECs to create HUVEC-Fluc. The dominant-negative caspase 3 [15, 36] with a key cysteine mutation in the catalytic domain of caspase 3 (C163A) was also obtained from Prof. Chuan-Yuan Li's laboratory. Live, replication-deficient recombinant lentiviral vectors expressing exogenous genes were packaged in 293T cells following manufacturer's instructions. HUVEC-Fluc, HT-29 CASP3DN were acquired through lentivirus infection and puromycin selection at 3 μg/ml.

### Endothelial cell growth measurement *in vitro* and *in vivo* with bioluminescence imaging and fluorescence imaging

*In vitro*, HUVEC-Fluc (100-500) were seeded onto a larger number (2-2.5 × 10^5^) of differentially irradiated HT-29 cells (feeder cells) within 24 hours since irradiation in 24-well plates or hanging cell culture inserts of 0.4 μm pore size (PIHT12R48; Millipore, MA, USA). Culture medium was replaced with fresh 2% FBS DMEM every 2 days. After a coculture period of 9-14 days, to measure luciferase activity, we added D-Luciferin potassium (bc219; Synchem UG & Co. KG, Felsberg/Altenburg, Germany) diluted in PBS (0.15 mg/ml) into each well before bioluminescence imaging.

*In vivo*, male nude mice (4-6 weeks old) were used. We injected a small number (1 × 10^5^) of HUVEC-Fluc either alone or mixed with a large number (1 × 10^6^) of 10 Gy-irradiated HT-29 cells. Cells were resuspended in PBS and 100 μl cell suspension was injected subcutaneously into hind legs of nude mice. Before non-invasive bioluminescence imaging, mice were injected intraperitoneally with 150mg/kg D-luciferin potassium dissolved in deionized water (30 mg/ml) and then anesthetized with continuous flow of isoflurane. To make bioluminescent signals from different batches of mice comparable, we kept the time between D-luciferin potassium injection and imaging at 10 minutes.

Bioluminescence imaging machines used in this study were NC100 instrument (Berthold Technologies GmbH & Co. KG, Bad Wildbad, Germany) and SPECTRAL Ami X (Spectral Instruments Imaging, Tucson, AZ, USA). After images were taken, we employed manufacturer-supplied software to process images for quantitative data.

For GFP fluorescence imaging of live HUVEC-Fluc *in vitro*, a confocal laser scanning microscope (Leica Microsystems, Mannheim, Germany) was used.

### Conditioned medium collection

Conditioned medium was prepared mainly as previously described [50]. In brief, an equal number of tumor cells were seeded in cell culture dishes overnight. Culture medium was replaced by complete medium before irradiation. 48 hours after irradiation, culture medium was harvested, centrifuged at 2500 rpm for 5min, filtered with 0.22 μm filter unit (Merck KGaA, Darmstadt, Germany) and stored at −80°C until use.

### HUVEC migration assay

HUVEC migration was performed with hanging cell culture inserts of 8 μm pore size (PIEP12R48; Millipore) for 24-well plates as previously described [50]. Mainly, 600 μl conditioned medium was placed into the lower chamber of every well and 200 μl serum free DMEM containing HUVECs (5 × 10^4^) added on the top of inserts. After 6 hours, cells staying in the inserts were removed gently with cotton swabs. Transmigrated HUVECs were fixed with 4% paraformaldehyde and then stained with crystal violet. Number of migratory HUVECs was measured by counting in five random fields under microscope.

#### Immunofluorescence (IF) and immunohistochemistry (IHC) analysis

Xenograft tumor and matrigel plug sections were deparaffinized with xylene and rehydrated with addition of ethanol. Heat-induced antigen retrieval was performed in Tris-EDTA buffer. Tissue sections were subjected to 3% hydrogen peroxide to remove endogenous peroxidase activity and then blocked with blocking buffer (P0102; Beyotime, China). Slides were incubated with primary antibodies against CD31 (sc-1506; Santa Cruz Biotechnology, Dallas, Texas, USA), VWF (sc-14014; Santa Cruz Biotechnology), CD34 (#3569; Cell Signaling Technology, MA, USA), and GFP (ARH2068; Antibody Revolution, San Diego, CA, USA) at 4°C overnight. Sections were washed three times for 5 minutes each time. For immunofluorescence analysis, fluorescein-labeled secondary antibodies (20014; 20106; Biotium, Hayward, CA, USA) were incubated at room temperature for 1 hour. Before immunofluorescence analysis with a confocal microscope, cells were counterstained with DAPI. For immunohistochemistry analysis, Real Envision Detection Kit (GK500710; Gene Tech, Shanghai, China) was used and signals were visualized through the diaminobenzidine reaction.

### Flow cytometric analysis

Cell apoptosis was analyzed with FITC Annexin V apoptosis detection kit (556547; BD Pharmingen^TM^, San Diego, CA, USA). Procedures were carried out according to the technical data sheet from the kit. Briefly, cells were trypsinized, washed, and resuspended in binding buffer. After FITC Annexin V and PI staining for 15 minutes, apoptosis was detected using Accuri C6 Flow cytometer (BD Biosciences, CA, USA).

### Western blot analysis

Western blot analysis was performed mainly as previously described [51]. Proteins were extracted, separated by gel electrophoresis and transferred to polyvinylidene difluoride (PVDF) membranes. The membranes were blotted with primary antibodies for β-actin, caspase 3, cleaved caspase 3, HA-tag, GAPDH and p-Akt (#4967; #9665; #9661; #3724; #5174; #4060; Cell Signaling Technology) and then secondary antibodies (Jackson ImmunoResearch, PA, USA). Finally, Immobilon^TM^ Western Chemiluminescent HRP Substrate (P90719, Millipore Corporation, Billerica, MA, USA) was utilized to visualize signals on PVDF membranes.

### Confocal microscopy for cleaved caspase 3

HT-29 cells were seeded into 35 mm glass bottom dishes with 10 mm micro-well (D35-10-1-N; *In Vitro* Scientific, Sunnyvale, CA, USA) overnight. Having undergone 10 Gy irradiation, cells were rinsed with PBS and fixed with 4% paraformaldehyde for 15 minutes. Subsequently, cells were permeabilized with 0.3% Triton X-100 diluted in PBS for 15 minutes and then blocked with blocking buffer (Beyotime) for 1 hour. Then, cells were incubated with primary antibody against cleaved caspase 3 (Cell Signaling Technology) at 4°C overnight. After washed with PBS three times for 5 minutes each time, cells were subjected to fluorescent secondary antibody (20125; Biotium) at room temperature for 1 hour. Before confocal microscopy analysis, cells were counterstained with DAPI.

### Tube formation assay

Tube formation assay was performed in 48-well plates. Matrigel (BD Biosciences) was thawed at 4°C overnight on ice. 150 μl matrigel was coated into each well and incubated at 37°C for 1 hour. Once matrigel solidified, 500 μl conditioned medium containing 6 × 10^4^ HUVECs was added on the matrigel surface with pre-cooled pipettes. After 6 to 8 hours, tube formation was photographed with inverted phase contrast microscope. For data analysis, number of formed loops was calculated.

### Matrigel plug assay

For matrigel plug assay, male nude mice (4-6 weeks old) were used. 500 μl matrigel mixed with 2 × 10^6^ either HT-29 or HT-29 CASP3DN cells treated with 10 Gy irradiation was implanted subcutaneously into either flank of mice, respectively. 8 days after injection, mice were sacrificed and plugs were harvested. Skin vasculature adjacent to plugs was photographed.

### Tumor xenograft experiment

Male nude mice (4-6 weeks old) were used. 2 × 10^6^ either HT-29 or HT-29 CASP3DN cells were resuspended in 100 μl PBS and injected subcutaneously into either flank of mice, respectively. Tumor size was measured every week until 6 weeks and tumor volume (V) was calculated using the formula: V = 0.5 × length × width^2^. Micro-CT (PerkinElmer, MA, USA) was performed before sacrificing mice.

### Quantitative real-time PCR

Total RNA was extracted from cultured cells using RNAsimple Total RNA Kit (Tiangen, Beijing, China). The reverse transcription reaction was carried out using 1 μg of total RNA with PrimeScript™ RT Master Mix (Takara, Otsu, Shiga, Japan). Quantitative real-time PCR was performed with SYBR^®^ Premix Ex Taq™ II (Takara). Briefly, 1 μl cDNA was added per 20 μl reaction with sequence-specific primers and SYBR^®^ Premix Ex Taq™ II. The specific forward and reverse primers of VEGF-A (NM_001171627) were 5′-AGGGCAGAATCATCACGAAGT-3′ and 5′-AGGGTCTCGATTGGATGGCA-3′. For GAPDH (NM_001256799), the specific forward and reverse primers were 5′-GGAGCGAGATCCCTCCAAAAT-3′ and 5′-GGCTGTTGTCATACTTCTCATGG-3′. VEGF-A gene expression was calculated according to the 2^−ΔΔCt^ method.

### ELISA

HT-29 cells were seeded onto 60 mm cell culture dishes overnight. 4 hours before irradiation, HT-29 cells were administrated with 15 μmol Z-DEVD-FMK (14414; Cayman Chemical, MI, USA) or vehicle control. 1 day and 2 days after irradiation, cell culture supernatant was collected, centrifuged and stored at −80°C until use. Human VEGF Valukine ELISA Kit (VAL106; R&D Systems, MN, USA) was used to evaluate the VEGF-A concentration of cell culture supernatant. Procedures were conducted in strict accordance with the manuscript in the kit.

### Other drugs used

Ranibizumab, also known as Lucentis, was available from Novartis (Switzerland). Ki8751 was available from Selleckchem (TX, USA).

### Ethics

All animal procedures were approved by the Animal Care Committee at Shanghai General Hospital. We made every effort to reduce the number of animals used and their discomfort during experiment.

### Statistical analysis

All data were presented as mean ± SEM. For parameter tests, statistical significance was evaluated with unpaired 2-tailed student's *t* test. For nonparametric tests, Mann-Whitney *U* test was used to analyze levels of significance. In all cases, the difference was considered statistically significant, if *p* value was less than 0.05.
